# Bushen-Yizhi formula ameliorates mitochondrial dysfunction and oxidative stress via AMPK/Sirt1 signaling pathway in D-gal-induced aging rats

**DOI:** 10.1186/s13020-023-00755-3

**Published:** 2023-05-11

**Authors:** Yanfang Liao, Yiyi Lai, Huilin Xu, Li Gao, Xiaomei Fu, Xue Wang, Qi Wang, Jiangang Shen, Jiansong Fang, Shuhuan Fang

**Affiliations:** 1 Science and Technology Innovation Center, Guangzhou University of Chinese Medicine, Guangzhou, 510405 People’s Republic of China; 2 Modern Research Center for Traditional Chinese Medicine, Shanxi University, Taiyuan, 030006 People’s Republic of China; 3 School of Chinese Medicine, the University of Hong Kong, Hong Kong S.A.R, People’s Republic of China

**Keywords:** Bushen-Yizhi formula, D-galactose-induced-aging rats, Metabolomics, Mitochondrial dysfunction, Oxidative stress, Inflammation, AMPK/Sirt1 signaling, Neurodegeneration

## Abstract

**Background:**

As a major risk factor for neurodegenerative diseases, aging has become a heavy health care burden worldwide. Age-related decline in mitochondrial function and oxidative stress is strongly associated with neurodegeneration. The previous study demonstrated that Bushen-Yizhi formula (BSYZ), a traditional Chinese medicine formula, is effective in reducing neurodegeneration.

**Methods:**

This study is the first to investigate the effect of BSYZ on D-gal-induced learning memory in rats. Secondly, the potential metabolic mechanism of BSYZ was explored by ^1^H-NMR metabolomics analysis. Then based on the comparison of differential metabolites implied that BSYZ ameliorated mitochondrial dysfunction through choline metabolic pathway in D-gal-treated rats. Finally, pharmacological validation was conducted to explore the effects of BSYZ on D-gal-induced oxidative stress, neuroinflammation, and neuronal apoptosis.

**Results:**

Our data showed that BSYZ increased aspartate and betaine levels, while decreasing choline levels. Furthermore, BSYZ also increased the proteins level of CHDH and BHMT to regulate choline metabolic pathway. Meanwhile, BSYZ alleviated mitochondrial damage and oxidative stress, including enhanced ATP production and the ratio of NAD^+^/NADH, reduced the level of MDA, enhanced GSH and SOD activity, upregulated the expressions of p-AMPK, SIRT1 proteins. In addition, BSYZ downregulated the levels of inflammatory cytokines, such as TNF-α, IL-1β and IL-6, as well as suppressed Bcl-2 proteins family dependent apoptosis.

**Conclusion:**

BSYZ treatment effectively rescues neurobehavioral impairment by improving mitochondrial dysfunction, oxidative stress, neuroinflammation and neuroapoptosis via AMPK/SIRT1 pathway in D-gal-induced aging.

## Background

Aging is one of the major factors in neurodegeneration, involving Alzheimer's disease, Parkinson's disease, diabetes and cancer [1]. Previous studies have shown neurodegeneration involves widespread neuronal death, leading to impairments in learning and memory [2]. Mitochondria are considered the main source of cellular energy and play an important role in various age-related neurological diseases [3, 4]. With aging, mitochondrial dysfunction not only affects the formation of ATP, but also affects the imbalance of cellular redox state, leading to the occurrence of various neurodegenerative diseases [5]. High levels of ROS induce oxidative stress, and the resulting biomolecules disrupt neuronal homeostasis, ultimately leading to neuronal cell death [6].

The previous studies have shown that long-term chronic injection of D-gal exhibits features similar to natural aging [7], such as shortened lifespan, mitochondrial dysfunction [8], and increased oxidative stress [9], and is currently the most commonly used animal model to study brain aging. Notably, mitochondrial dysfunction leads to the production of reactive oxygen species (ROS) that triggers oxidative stress and inflammation, which are two major drivers of (aging) neurodegeneration [10]. However, mitochondrial DNA is also sensitive to ROS-induced oxidative damage. Hence, mitochondrial dysfunction as a cause and/or consequence of oxidative stress is one of the main drivers of these processes. AMPK is a key regulator of cellular energy homeostasis and metabolism, which plays a critical role in regulating mitochondrial function and preventing aging [11, 12]. Accumulating evidence suggests that activation of AMPK has a broad neuroprotective role in AD [13, 14]. Moreover, AMPK activation increases intracellular NAD^+^ concentration and activates Sirt1 [15, 16]. Sirt1 is an NAD^+^-dependent sirtuin, which has been reported to extend the lifespan of numerous organisms including yeast [17], flies and nematode [18], Caenorhabditis elegans [19] and mice [20]. The increase of Sirt1 levels and/or activity has been shown to have beneficial effects in neurodegeneration including Alzheimer’s disease, Parkinson’s disease and Huntington’s disease [21]. Likewise, activated AMPK and Sirt1 induce mitochondrial biogenesis [22] and inhibit other pathological responses.

Historically, Traditional Chinese medicine (TCM) herbal decoctions had been made by means of way of exclusive technique of special combinations of one of type herbs into one formula, and have been used as drugs or health dietary supplements in China for hundreds of years [23]. Among thousands of TCM formulas, BSYZ is a TCM decoction consisting of Cnidium, Cortex Moutan, Ginseng, Polygonum multiflorum, Lycium barbarum, Ligustrum lucidum. Our preceding studies have demonstrated that BSYZ has extensive neuroprotective functions in neurodegeneration, such as Alzheimer's disease, Parkinson's disease [24, 25]. However, the metabolomic mechanism of BSYZ remains unclear in neurodegeneration. In this study, we investigated the mechanism of BSYZ on D-gal-induced aging used the ^1^HNMR-based metabolomics. Additionally, we evaluated the neurobiological modifications exhibited with the aid of BSYZ in the aging model triggered via D-gal, which includes changes in mitochondrial dysfunction, oxidative stress, neuroinflammation and apoptosis.

## Materials and methods

### Experimental animals

Sprague Dawley male rats (2 months of age,180–220 g) were purchased from Vitong Lihua Experimental Animal Co., Ltd. (Zhejiang, China; Animal certificate number: SCXK-(Zhejiang) 2019–0001). All rats were acclimated for 1 week under 12-h light/dark conditions at 23–25 °C, 60 ± 10% humidity, and were provided with free water and food. All procedures were approved and carried out under the guidance of the Animal Ethics Committee of Guangzhou University of Traditional Chinese Medicine (approval number 20200717003).

### Experimental design and drug administration

**Fig. 1:**
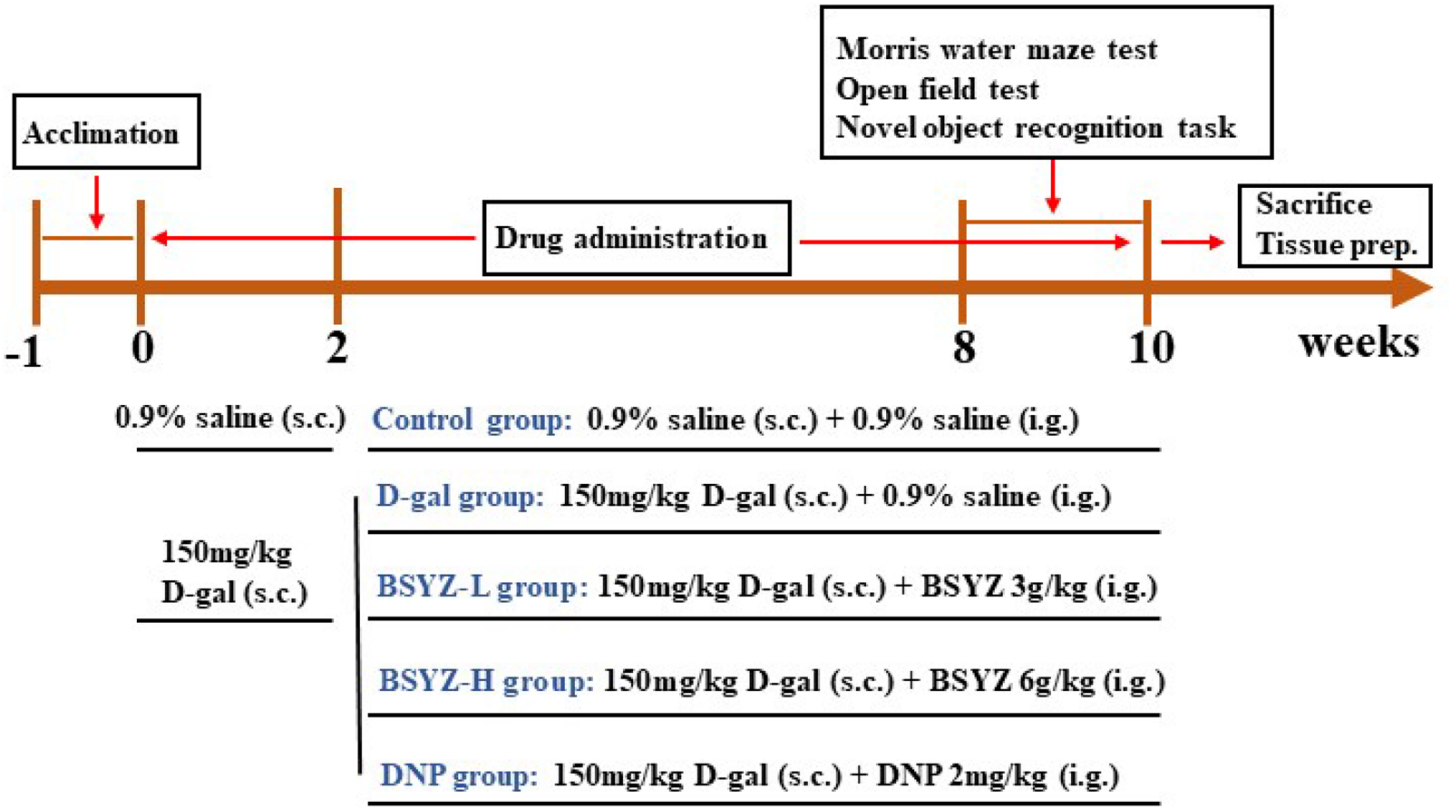
Experimental protocol of the study. Rats were randomly divided into 5 groups after one week of adaptive feeding. Expect for the control group, all animals received subcutaneous injection of D-gal (150 mg/kg) for 10 weeks. The rats in the BSYZ-L, BSYZ-H and DNP groups received oral gavage every day in the third week, and last into the tenth week. The behavioral tests, including morris water maze, open field test and novel object recognition task) were performed during the ninth and tenth weeks. After the behavioral tests, the animals were sacrificed and brain tissue was collected for experimental analysis

The experimental rats were randomly divided into the following five groups (n = 10 rat/group): Control (con) group (0.9% saline s.c. + 0.9% saline i.g.), D-galactose (D-gal) group (150 mg/kg/day s.c. + 0.9% saline i.g.), low dose Bushen-Yizhi Formula (BSYZ-L) group (D-gal 150 mg/kg/day s.c. + BSYZ 3 g/kg/day i.g.), high dose Bushen-Yizhi Formula (BSYZ-H) group (D-gal 150 mg/kg/day s.c. + BSYZ 6 g/kg/day i.g.), Donepezil (DNP) group (D-gal 150 mg/kg/day s.c. + DNP 2 mg/kg/day i.g.). After one week of adaptive feeding, all rats treated with D-gal s.c., while the con group was given 0.9% saline i.g. Next, starting in the third week of the trial, rats received 0.9% saline, BSYZ, or DNP orally for 8 consecutive weeks. The experimental protocol is showed in Fig. 1.

### Animal behavioral test

#### Morris water maze test (MWM)

The Morris water maze pool is 150 cm in diameter and 60 cm high. The tank was filled with 40 cm high water, and an appropriate amount of non-toxic food stain (Mixol, Germany) was added to the water so that the platform and the bottom of the pool were invisible to rats. A 10-cm-diameter platform was placed on the target quadrant. An automated camera was fixed above the center of the maze to monitor and record the animal's route during behavioral tests. The day before training, the rats underwent an acclimation period, allowing them to swim in the pool and rest on the platform for 30 s. During training, the rats were placed in one of the quadrants and allowed to search for underwater platforms. Animals that did not find the platform within the allowed time were physically guided and placed on the platform for 10 s before being removed. On day 6 after training, a probe test was performed without a platform in the pool to check memory retention. The animals were allowed to swim from one of the quadrants for 60 s, and the number of times the rats crossed the quadrant and the time they spent in the formal platform area were measured.

#### Open field test (OFT)

The rats were tested of locomotor activity in OFT, which is a common measure of general activity and exploratory behavior in mice and rats. The OFT instrument (rectangular) interior walls are painted black, the dimensions are 100 × 100 cm, and the walls are 40 cm. The bottom is divided into 25 smaller rectangular units. The animals were acclimated for 30 min in a quiet, dark laboratory before the experiment began. Each rat was placed in the center and allowed to explore freely for 5 min. At the end of each measurement, the OFT apparatus was cleaned with 70% alcohol and dried after each test to avoid the effect of odor on the next rat.

#### Novel object recognition task (NOR)

Using the field test box, the non-spatial memory ability was tested by taking advantage of the rat's instinct to touch and explore novelties. On the first day of the experiment, the animals were placed in the experimental box and familiarized with the environment for 5 min. The next day, two identical objects were placed symmetrically in the experimental box. The distance between the two objects is 10 cm from the side and back walls of the box. Record the total time the rat spends exploring both objects within 5 min. After each probe, the secretions should be cleaned with 75% alcohol. On the third day, one of the familiar objects was replaced by another new object of similar size but different shape and color. Time spent exploring novel and familiar objects was recorded. Object detection is defined as placing the mouse's nose within 3 cm of the object, or sniffing, licking, touching the object with its front paws, and sniffing at the same time.

### Brain tissue collection and sample preparation

After assessment of cognitive ability, rats were anesthetized with pentobarbital sodium (30 mg/kg). For biochemical analysis, they immediately removed the brain, carefully dissected the hippocampus and cerebral cortex regions, and snap-frozen in liquid nitrogen. For immunostaining and morphological analysis, saline was perfused transcardially, followed by 4% paraformaldehyde (PFA). Brain tissue was carefully removed, immersed in water, fixed in ice-cold PFA at 4 °C for 72 h, then rinsed with phosphate-buffered saline (PBS), placed in 30% sucrose solution for 48 h, and frozen in OCT complexes. Serial 20 um coronal tissue sections were cut on a cryostat (Leica, Nussloch, Germany) and then thawed on gelatin-coated slides.

### Sample collection and preparation for ^1^H-NMR spectroscopy

After cortical samples were thawed at 4 °C, 200 mg of cortical samples were homogenized by adding 600 μL methanol and 300 μL distilled water in an ice bath. After centrifugation at 4 °C and 13,000 r/min for 20 min, we pipet 500 μL of the supernatant into a 1.5 mL EP tube and use a vacuum drying concentrator to dry the samples. Then, the dried samples were dissolved in 600 μL of mixed phosphate buffer (0.2 mol/L Na2HPO4, NaH2PO4, 0.005% TSP, pH 7.4), centrifuged at 4 °C, 14,000 r/min for 10 min, and 550 μL of supernatant was precisely collected, and transferred into a 5 mm NMR tube for NMR analysis.

### NMR analysis and data preprocessing

The ^1^H-NMR spectra were collected on a Bruker 500 MHz Avance III NMR spectrometer (Bruker, Germany). The spectra were recorded using a NOSEY pulse sequence at 298.15 K (25 °C), and the following parameters were used: FID resolution of 0.188 Hz; pulse time 14 μs; sampling time 2.654 s; delay time 1.0 s; sampling interval 40.5 μs and sampling point 65,536.

All acquired data were Fourier transformed using MestReNova software (Mestrelab Research, Santiago de Compostella, Spain). The obtained spectra were manually phased and the baseline was corrected to calibrate peak positions with a TSP of zero. Subsequently, the spectra were integrated by chemical shift interval δ 0.50—9.00 segmented with δ 0.01 as integration area, water peaks removed (δ 4.72—δ 5.2), and data normalized for further analysis.

### Betaine assay

To verify the result of nontarget metabolomics, the content of betaine was determined. As a methyl donor, betaine promotes a variety of biological processes. The betaine assay kit (Heifei Lai Er Bio-Tech Hefei, China) was used to measure the level of betaine in the brain. According to the manufacturer’s instruction, we weighed about 0.2 g of the oven dried sample, added 1 ml of extraction solution, and placed at 60 °C extraction for 30 min, during constant shaking. The supernatant was obtained after centrifuging for 15 min at 25 °C after adding 3 mg of the reagent 4 for shocking thoroughly. The methanol was volatilized clean in a 70 °C oven, then diluted with double distilled water to a volume of one ml. By adding the sample and reagent in accordance with the operating procedures, mix thoroughly. The microplate reader was preheated for more than 30 min, the wavelength was adjusted to 525 nm, and the OD value of each tube was measured. Finally, the concentration is calculated by the formula.

### SOD, MDA and GSH assays

Firstly, the tissue was rapidly homogenized in ice-cold PBS (9 times of tissue weight) using a homogenizer. After centrifugation at 4500 g for 15 min at 4 °C, the supernatant was collected to determine the protein concentration using a protein assay kit (Beyotime, China). For measuring SOD, the Superoxide Dismutase Detection Kit (Nanjing Jiancheng Bioengineering Institute, Nanjing, China) was used. According to the manufacturer's instruction [26], the assay was carried out. GSH serves as the body's most significant non-enzymatic antioxidant and scavenges free radicals. As directed by the manufacturer's instruction, we employed a Reduced glutathione assay kit (Nanjing Jiancheng Bioengineering Institute, Nanjing, China) to ascertain the level of GSH present in brain tissue. GSH may form a yellow molecule when it reacts with dithiodinitrobenzoic acid (DTNB), which enables colorimetric quantitative measurement of GSH content at 405 nm. MDA is a marker of lipid peroxidation. In accordance with the manufacturer's instruction from the Malondialdehyde assay kit (Nanjing Jiancheng Bioengineering Institute, Nanjing, China), we employed thiobarbituric acid (TBA) to measure the MDA concentration [26, 27]. Finally, a colorimetric test at 532 nm was performed on the supernatant in each tube.

### ATP assay

The ATP assay kit (Nanjing Jiancheng Bioengineering Institute, Nanjing, China) measures the level of adenosine 5'-triphosphate (ATP), the most fundamental carrier of energy conversion in living things. The tissue was precisely weighed, and a 10% homogenate was created by adding 9 L of cold, double-distilled water to an ice water bath (portions were removed and centrifuged at 3500 rpm for 10 min to measure protein concentration of the supernatant). The homogenate was submerged in boiling water for 10 min, extracted, and subjected to a 1-min mixed extraction procedure. Following a 10-min centrifugation period at 3500 g, the supernatant was collected and loaded as manufacturer's instruction. Finally, it was allowed to stand for 5 min at room temperature after mixing, and the absorbance value of each tube at 636 nm wavelength was measured.

### NAD^+^/NADH Assay

The NAD^+^/NADH assay kits (WST-8, Abbkine Scientific Co., Ltd, California, America) were provided to measure the NAD^+^ and NADH contents following the manufacturer’s instructions. We weighed 20 mg of rat brain tissue and added 100 µL of NAD Extraction Buffer or NADH Extraction Buffer to the homogenized samples, which were used to extract NAD and NADH for further analysis. Briefly, the absorbance at 450 nm was measured immediately after adding the sample to the 96-well plate according to the instructions, followed by incubation at room temperature for 30 min, and then measuring the absorbance at 450 nm. Finally, The NAD^+^/NADH ratio was calculated according to the formula.

### TNF-α, IL-1β and IL-6 ELISA kit assays

Before detection, cortical and hippocampal tissues were homogenized as before. TNF-α, IL-1β and IL-6 ELISA Kit Assays (Nanjing Jiancheng Bioengineering Institute, Nanjing, China) were detected according to the manufacturer’s recommendations.

### Nissl staining

After preparing coronal tissue sections of brain tissue as described above, which were fixed with 4% paraformaldehyde for 10 min, and then rinsed with distilled water 3 times for 2 min each time. The sections were stained with Nissl's stain solution (Beyotime Biotechnology, Shanghai, China) for 10 min in a 37 °C oven. After 10 min, rinse both sides with distilled water twice and then perform the following operations: Dehydrated in 95% ethanol for 2 min, then switch to fresh 95% ethanol for another 2 min. Dimerized with xylene for 5 min, then dimerized with xylene for another 5 min with fresh xylene. Finally, the sections were covered with rhamsan gum. Images were acquired with microscope (NICON Eclipse 80i, Nicon, Japan).

### TUNEL apoptosis assay kit

The apoptosis of nerve cells in D-gal-induced rats was saaessed by TUNEL staining (Beyotime Biotechnology, Shanghai, China). In summary, the frozen sections were fixed with 4% paraformaldehyde and then conducted with 0.25% Triton-X 100. Then, the sections were washed with PBS, and incubated in the TUNEL reaction solution for 1 h at 37 °C in humidified chamber in the dark, after which DAPI was used to stain the nucleus for 10 min. Finally, images were recorded under an fluorescence microscope (DMI8, Leica, Germany).

### Immunoblotting and immunofluorescence

Western blot analysis was performed according to the previous method in our laboratory. The bicinchoninic acid (BCA) method (Beyotime, China) was used to quantify the protein in cerebral infarct hemispheres or astrocytes. Protein samples were separated by SDS-PAGE and transferred to polyvinylidene fluoride (PVDF) membranes (Millipore, Billerica, MA). The membrane was blocked with 5% milk for 2 h at room temperature, incubated with the target protein-specific primary antibody overnight at 4 °C, and then incubated with the secondary antibody for 2 h at room temperature. Digital images of protein bands were acquired using Chemidoc XRS (Bio-Rad, Hercules)., CA, USA) and Bio-Rad Image Lab 5.2.1 software (Bio-Rad Labs, California, USA) for quantification.

For immunofluorescence, the fixed brain slices were rinsed 3 times with PBS (5 min/time). Treated with proteinase K for 5 min and rinsed twice with f-PBS for 5 min each. It was then blocked with 5% goat serum (0.3% Triton X-100 diluted in PBS) for 30 min at room temperature and then rinsed with PBS. Slides were incubated with primary antibodies overnight at 4 °C, followed by secondary antibodies with TRITC- or FITC-conjugated antibodies for 2 h at room temperature. After incubation, the secondary antibody solution was carefully removed, washed with PBS for 10 min, and counterstained with DAPI. Finally, the slides containing brain sections were covered with glass coverslips by using mounting media. Images were acquired with fluorescence microscope (DMI8, Leica, Germany).

### Statistical analysis

Combining published literature and NMR databases, including HMDB and BMRB, metabolites were identified by matching the signal peaks of the spectrum to individual metabolites. Multivariate analysis of NMR data was performed using SIMCA-P 13.0 (Umetrics, Switzerland), including principal component analysis (PCA), partial least squares discriminant analysis (PLS-DA), and orthogonal partial least squares discriminant analysis (OPLS-DA). The visualization of the model was implemented with SIMCA-P 13.0 and the quality of the model was assessed using the correlation coefficients R2X and Q2Y. The differential metabolites were obtained by s-plot plot combining VIP > 1, independent samples t-test p < 0.05, and analysis of variance to compare metabolite differences among multiple groups. Heatmap of differential metabolites among 3 groups by using the complete clustering method. The relative peak areas of differential metabolites among 3 groups were calculated using one-way ANOVA followed by post-hoc Tukey’s test.

For statistical analysis, all data are expressed as the mean ± standard error of the mean (M ± SEM). Statistical significance was determined by one-way ANOVA followed by the Tukey’s test. A value of P < 0.05 was considered to be statistically significant.

## Results

### BSYZ treatment enhanced learning, memory and spontaneous alteration behavior and ameliorated synaptic dysfunction in D-gal-induced rats

**Fig. 2:**
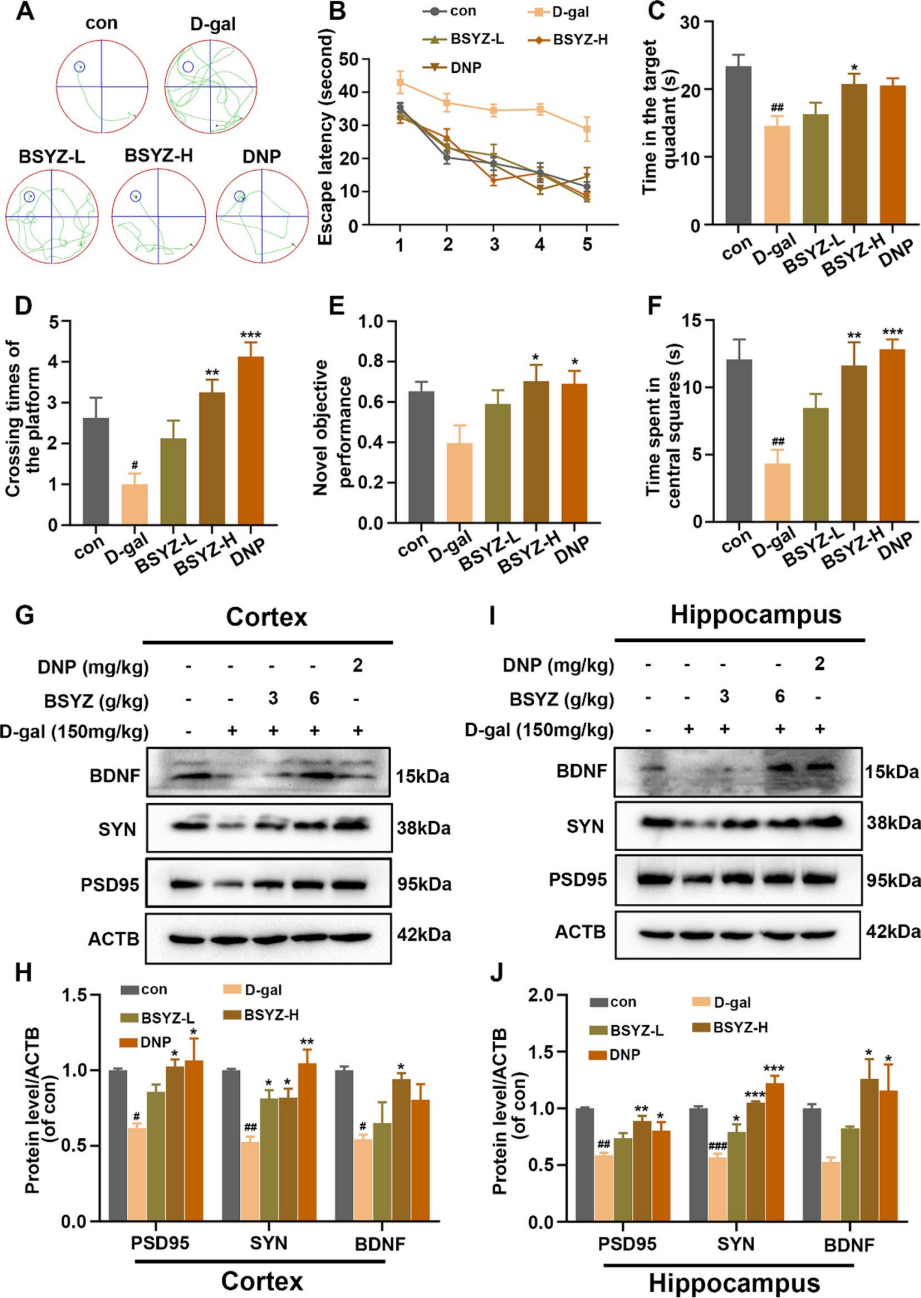
BSYZ inhabited D-gal-induced memory and synaptic dysfunction. **A** Representative trajectories during MWM training in rats. **B** Mean escape latency (sec) to reach the platform during 5 days of training. **C** The time spent in the target quadrant. **D** The number of target crossings. **E** Object recognition index. **F** The time spent in the central area. The expression of SYN, PSD95 and BDNF in the cortex **G**, **H** and hippocampus **I**, **J** by Western bolt. Data are expressed as mean (M ± SEM) and were evaluated by one-way ANOVA followed by post-hoc Tukey’s test. ^#^*P* < 0.05, ^##^*P* < 0.01 vs. the Con group. *P < 0.05, **P < 0.01, ***P < 0.001vs. the D-gal group

To analyze the effects of BSYZ on rat behavior and memory, we performed the Morris water maze (MWM) and the novel object recognition (NOR) tests. DNP was used as the standard positive control. In the MWM test, the escape latency of the D-gal model group was longer than that of the control group, indicating impaired spatial learning and memory ability. However, D-gal + BSYZ and D-gal + DNP groups significantly shortened the latency to reach the platform over training days compared the D-gal group (Fig. 2A, B). Similarly, during a probe test on a 5th day, we found that the number of platform crossings was significantly increased in the D-gal + BSYZ-H and D-gal + DNP groups compared to the D-gal treated group (Fig. 2C). In addition, the D-gal + BSYZ-H co-treated group spent more time in the target quadrant than the D-gal-treated group (Fig. 2D). In the NOR task, the effect of BSYZ treatment on D-gal-induced memory impairment was again explored. We found that D-gal reduced training and test object recognition indices compared to controls, while BSYZ as well as DNP treatment abolished the partial amnestic effect of D-gal (Fig. 2E). Thereafter, we performed the open-field test to analyze the effect of BSYZ on the spontaneous locomotion induced by D-gal. We found that D-gal-treated rats took less time to reach the central area than saline-treated (con) rats, indicating a deficit in spontaneous activity. However, the time to reach the central area was significantly increased in D-gal + BSYZ co-treated rats compared to D-gal-treated rats (Fig. 2F), suggesting that BSYZ ameliorates the spontaneous activity deficit in D-gal-treated rats.

Several studies have reported altered expression of synaptic proteins in D-gal-induced cognitive impairment [28]. To investigate the neurotoxic effect of D-gal and the neuroprotective effect of BSYZ on synaptic proteins, we analyzed the expressions of presynaptic protein (SYN) and postsynaptic protein (PSD95) by Western blotting. BDNF is a neurotrophic factor that plays a key role in learning and memory function by affecting synaptic plasticity [29]. Our results showed that SYN, PSD95 and BDNF were significantly reduced in the brains of D-gal-injected rats compared with control rats. Interestingly, BSYZ treatment significantly increased the expression levels of SYN, PSD95 and BDNF in the hippocampus compared to D-gal-treated group.

### BSYZ ameliorated metabolic abnormality in D-gal-induced rats

**Fig. 3:**
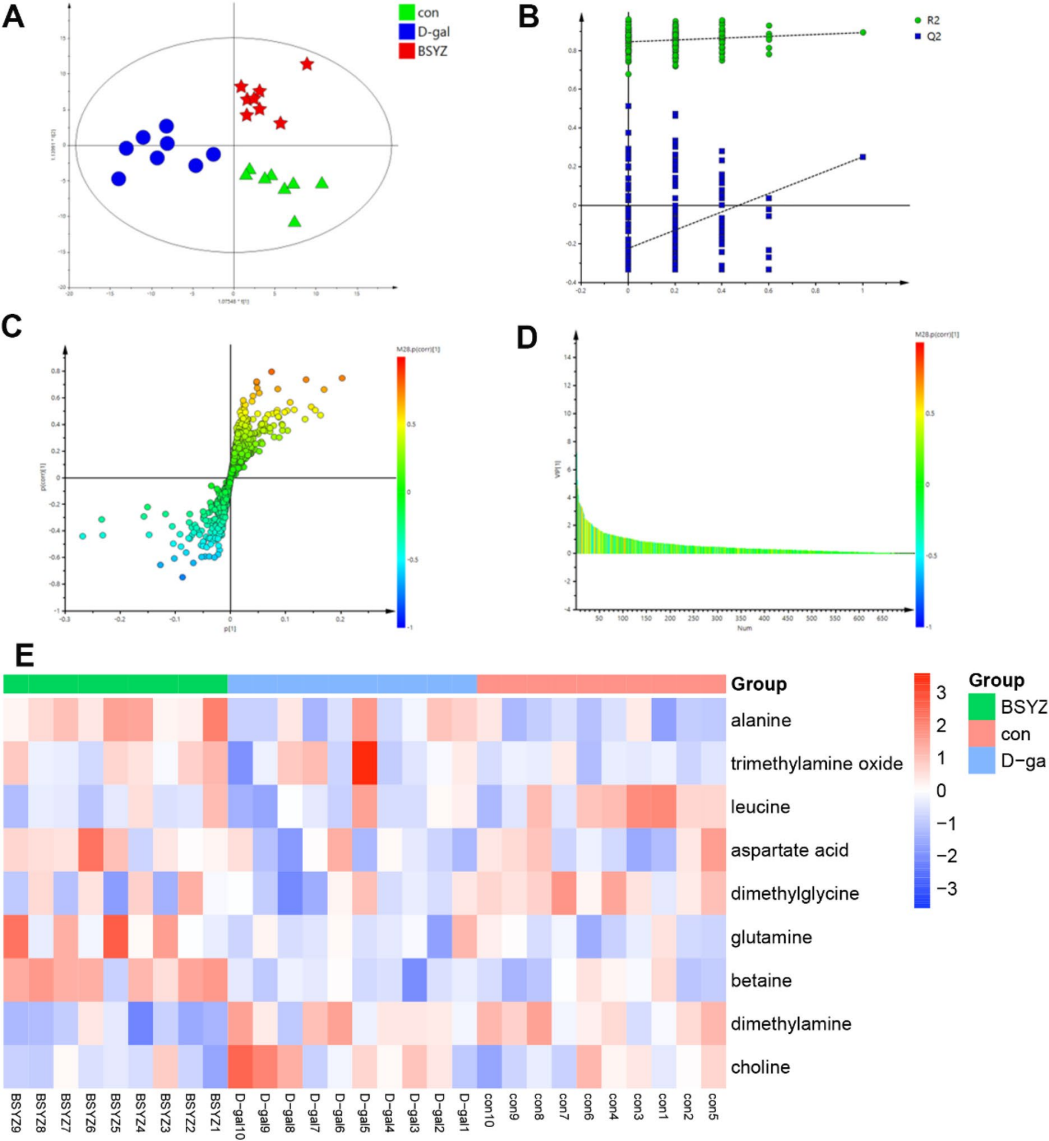
Multivariate statistical analysis of the metabolites in con, D-gal and BSYZ-H groups. **A** PLS-DA score plot; **B** PLS-DA validation plot, **C** S-plot plot, and **D** corresponding VIP values, compared between normal and model groups. **E** Heatmap of differential metabolites among 3 groups by using the complete clustering method

**Fig. 4:**
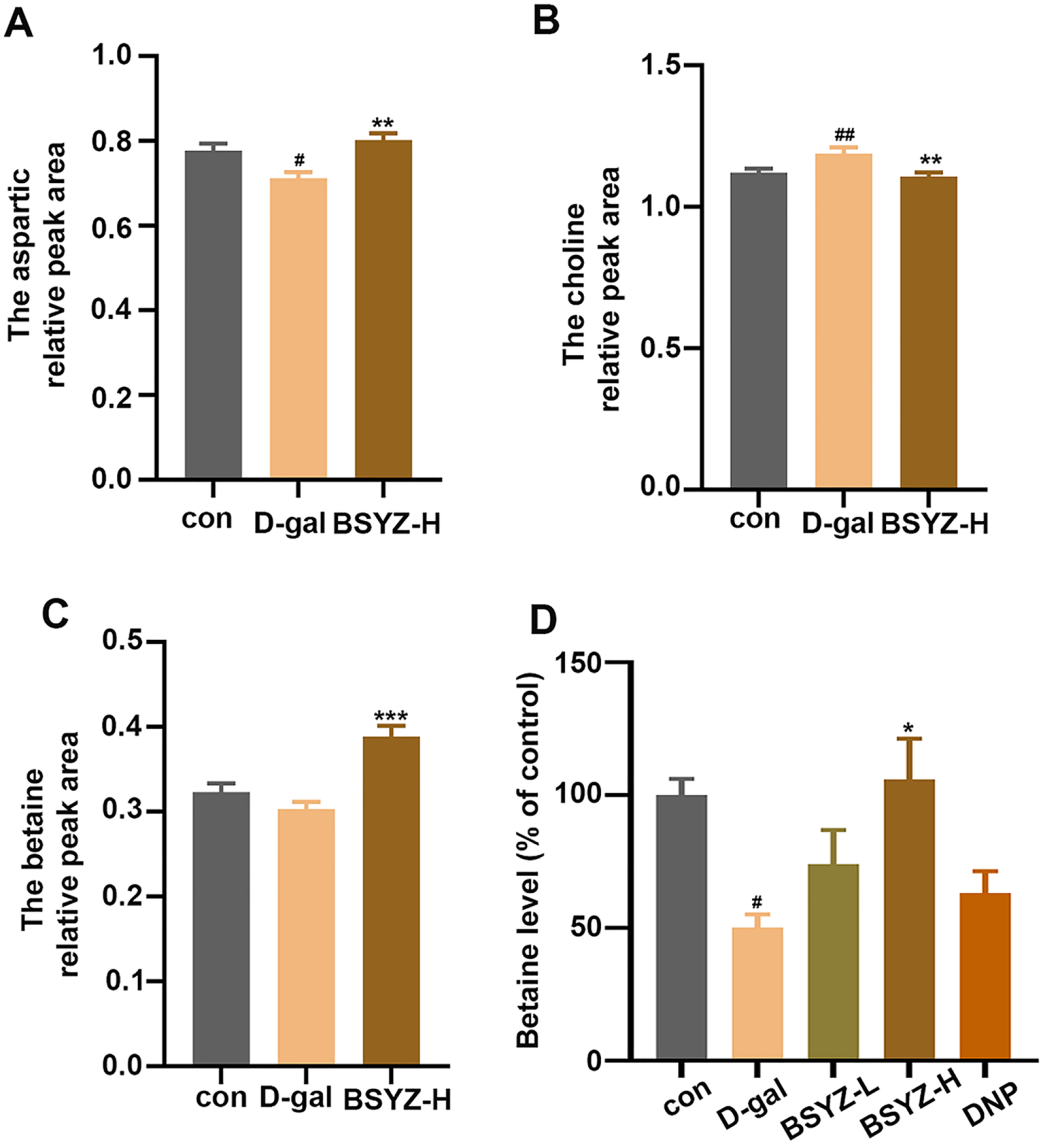
**A**–**C** Metabolomics analyzed the relative peak areas of aspartate, choline, and betaine. **D** The effect of BSYZ on betaine levels in the brain of experimental rats was detected by a kit. Data are expressed as mean (M ± SEM) and were evaluated by one-way ANOVA followed by post-hoc Tukey’s test. ^#^*P* < 0.05, ^##^*P* < 0.01 vs. the Con group. *P < 0.05, **P < 0.01, ***P < 0.001vs. the D-gal group

To identify the changes of endogenous metabolites in brain of D-gal-induced rats, we performed multivariate analysis of the NMR data. The results of partial least squares discriminant analysis (PLS-DA) (Fig. 3A) showed that the control group and D-gal group could be clearly separated, and the metabolic profiles after bsyz treatment were more similar to the control group. The cross-validation plot of PLS-DA indicated that the model was reliable with high predictive ability (Fig. 3B). The potential biomarkers were identified by the importance in projection values (VIP > 1) and S-plot (Fig. 3C, D). The Heatmap (Fig. 3E) demonstrated the differential metabolites among the 3 groups. Treatment with BSYZ obviously elevated the levels of aspartate and betaine. Besides, BSYZ markedly repressed the levels of choline (Fig. 4). To verify the result of betaine from the metabolomic study, we determinated the content of betaine in the cortex. As shown in the Fig. 4D, the level of betainle BSYZ-H group showed increased level of betaine.

### BSYZ improved mitochondrial function and choline metabolism pathway via upregulating AMPK/Sirt1 in D-gal induced model

**Fig. 5:**
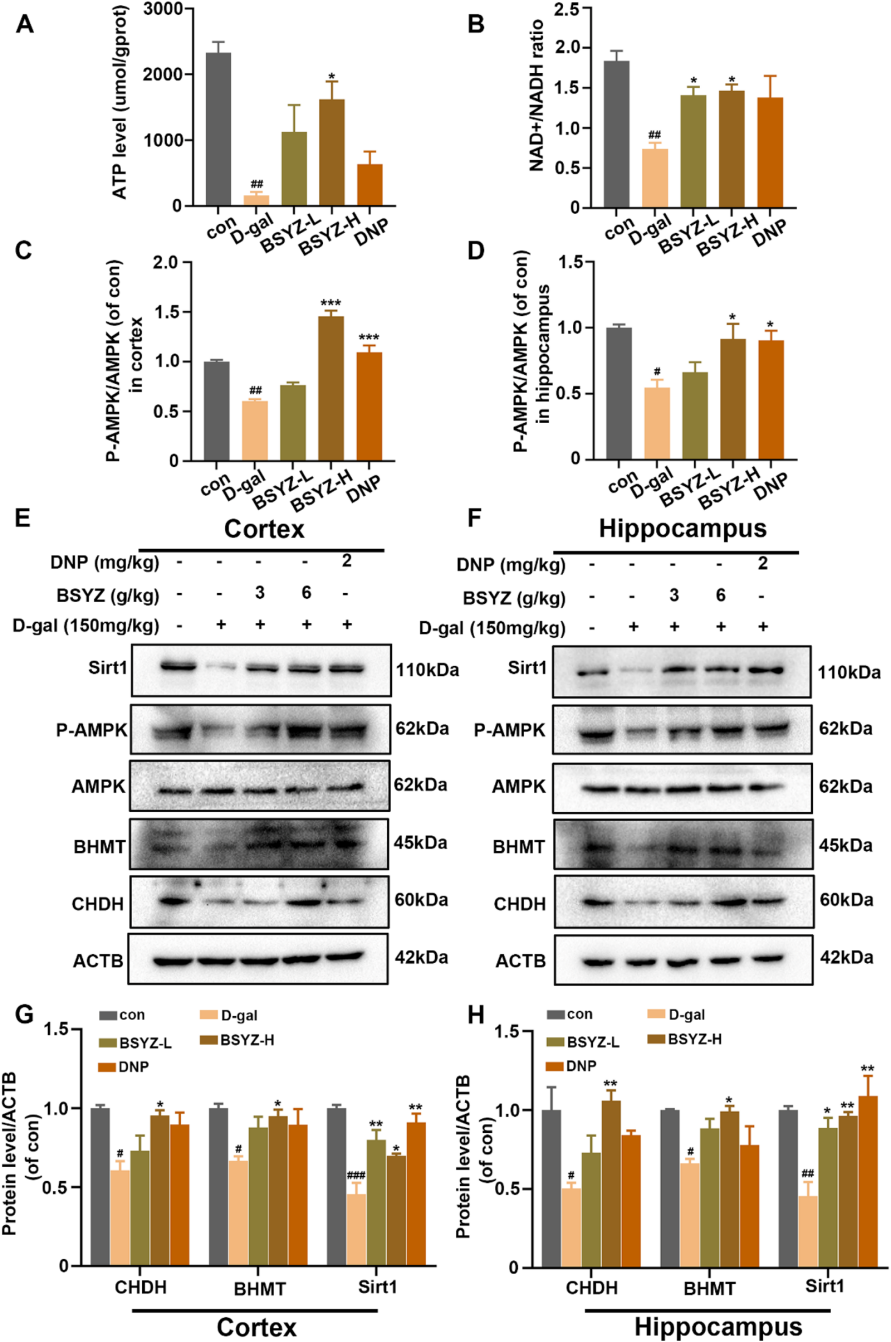
Treatment with BSYZ improved mitochondrial functions and choline metablism by upregulating AMPK/Sirt1 signaling, reduced by D-gal rats. **A**, **B** The representative histograms show the ATP levels and NAD^+^/NADH ratio in the cortex tissue lysate of the treated with BSYZ rats. **C**–**H** Weatern blot analysis of CHDH, BHMT, AMPK, P-AMPK and Sirt1in the cortex and hippocampus of experimental groups. Data are expressed as mean (M ± SEM) and were evaluated by one-way ANOVA followed by post-hoc Tukey’s test. ^#^*P* < 0.05, ^##^*P* < 0.01, ^###^*P* < 0.001 vs. the Con group. *P < 0.05, **P < 0.01 vs. the D-gal group

**Fig. 6:**
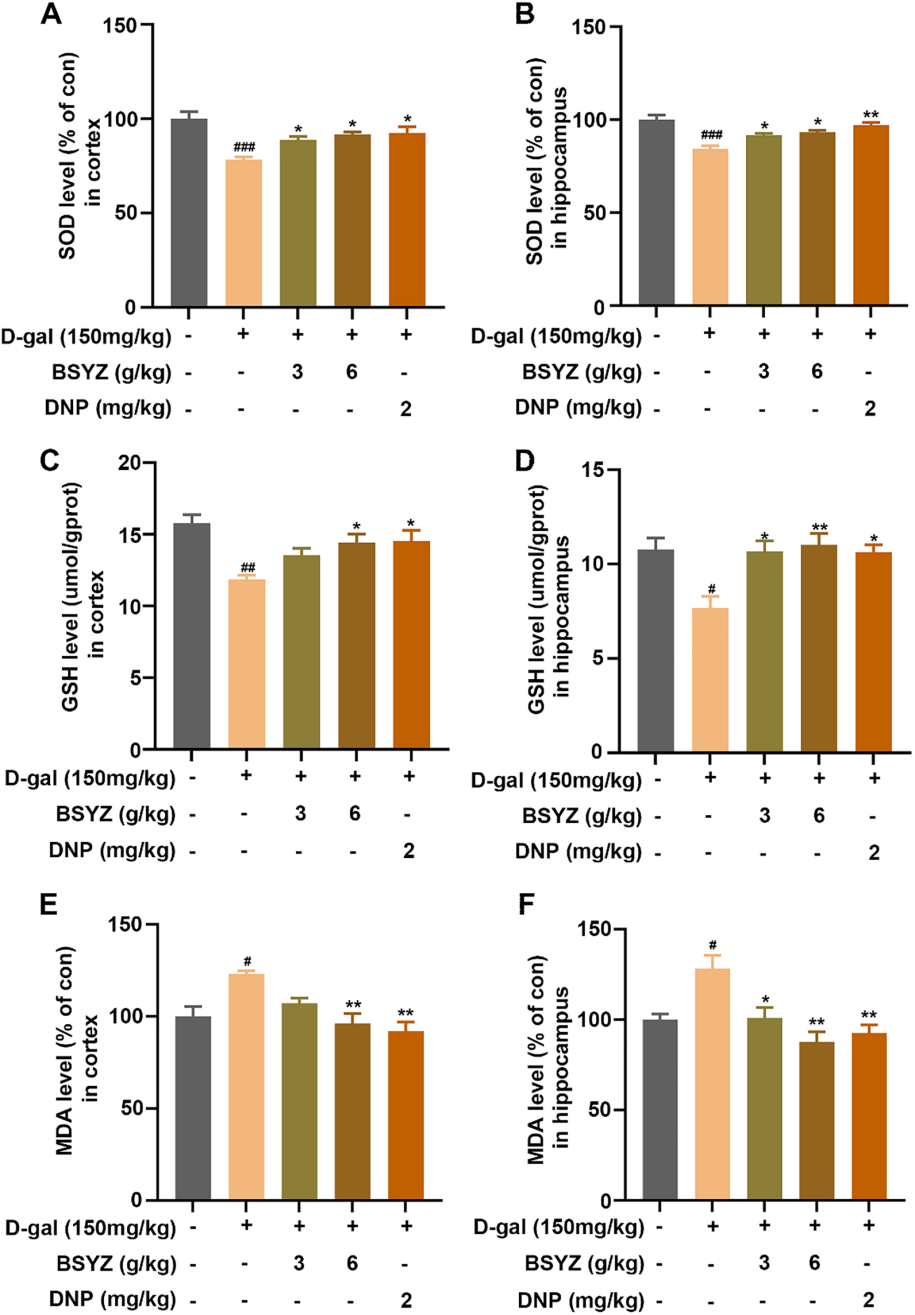
The effect of BSYZ on SOD (**A**, **B**), GSH (**C**, **D**) and GSH (**E**, **F**) levels in the cortex and hippocampus of D-gal group. Data are expressed as mean (M ± SEM) and were evaluated by one-way ANOVA followed by post-hoc Tukey’s test. ^#^*P* < 0.05, ^##^*P* < 0.01, ^###^*P* < 0.001 vs. the Con group. *P < 0.05, **P < 0.01 vs. the D-gal group

In the present study, we observed that treatment with BSYZ decreased choline and increased betaine levels in the cerebral cortex. Several studies shown that choline oxidation is initiated by choline dehydrogenase (CHDH) and further oxidized by betaine-aldehyde dehydrogenase to generating NADH and betaine [30]. Betaine is catalyzed by betaine-homocysteine methyltransferase (BHMT) to provide sufficient methyl groups to facilitate peer cysteine metabolism [31]. Interestingly, our immunoblotting results revealed that compared with the D-gal group, BSYZ-H treatment is significantly increased the expression of CHDH and BHMT in both cortex and hippocampus of the rats’ brain (Fig. 5). These findings indicate that BSYZ regulates choline metabolism in D-gal-induced model. What’s more, betaine is an endogenous AMPK agonist. AMPK acts as an energy sensor to maintain normal mitochondrial function [32]. Therefore, to determine whether BSYZ reverses D-gal-induced mitochondrial dysfunction, we examined the ATP and NAD^+^/NADH levels. The assays indicated a significantly increased in ATP and NAD^+^/NADH levels in cortex after treatment with BSYZ (Fig. 5A, B). Additionally, compared to saline treatment (con), the expression of P-AMPK and Sirt1 is markedly reduced in D-gal-treated group. However, compared with D-gal alone, D-gal + BSYZ co-treatment significantly increased the expression of P-AMPK and Sirt1 (Fig. 6). These results indicate that BSYZ ameliorates D-gal-induced P-AMPK-mediated mitochondrial dysfunction in the rat brains.

### BSYZ treatment attenuated D-gal-induced oxidative stress in the brains of rat

Previous studies have shown that mitochondrial dysfunction leads to ROS accumulation and cellular redox dyshomeostasis, and eventually promoted oxidative stress, which has detrimental effects on neuronal cells [33]. To ascertain effect of BSYZ against D-gal-induced oxidative stress, we performed superoxide dismutase (SOD), reduced glutathione (GSH) and malondialdehyde (MDA) assays in vivo. Our results shown that D-gal injection considerably elevated MDA production and reduced SOD and GSH generation. Conversely, BSYZ reduced elevated MDA level and increased SOD and GSH levels in the cortex and hippocampus of the rat brain (Fig. 6).

### BSYZ treatment ameliorated D-gal-induced neuroinflammation and glial cell activation in D-gal model

**Fig. 7:**
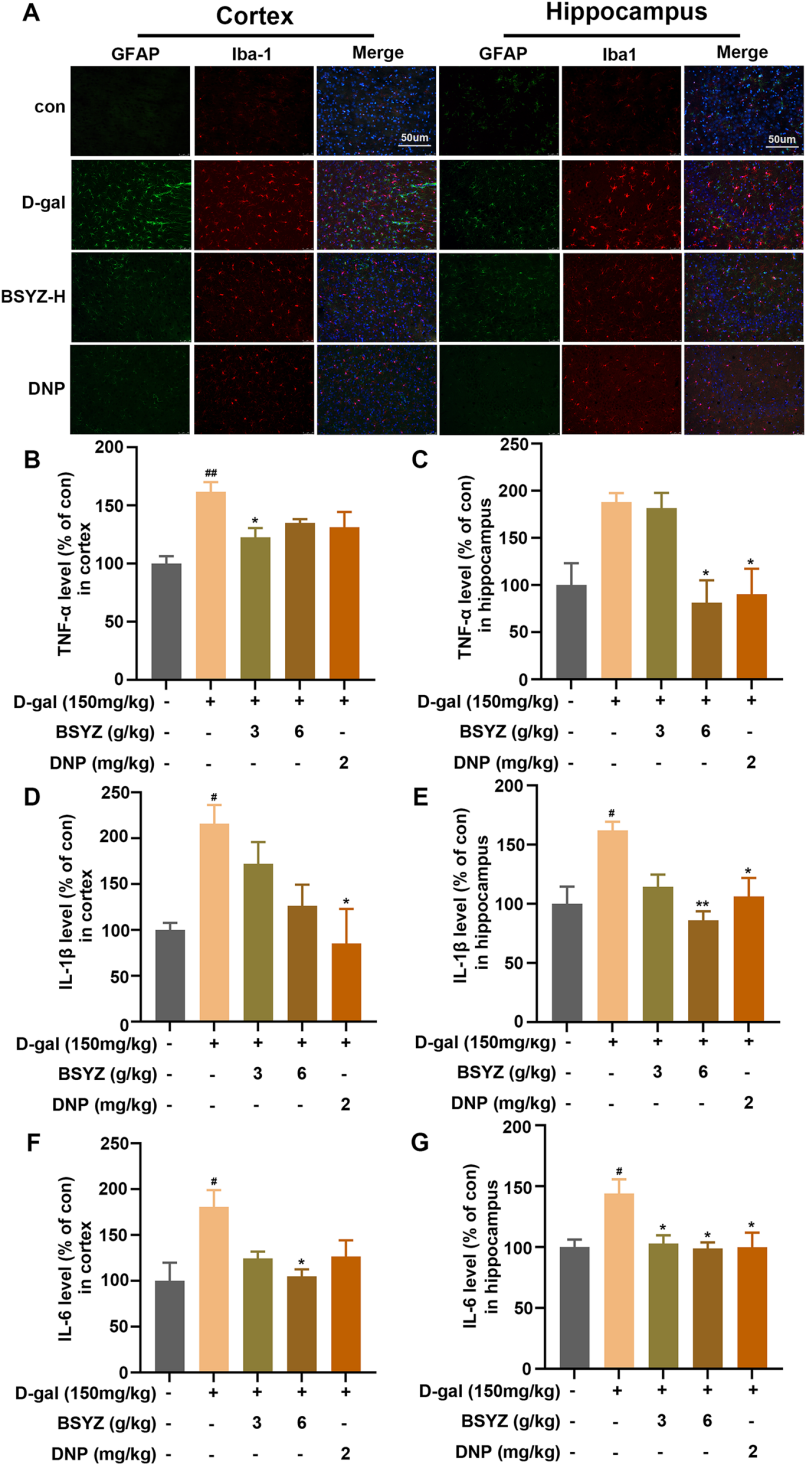
BSYZ inhibited D-gal-induced neuroinflammatory in the rat brain. **A** The immunofluorescence photomicrographs represent the immunoreactivity of GFAP and Iba1 in the cortex and hippocampus of rat. The Elisa analysis of TNF-α (**B**, **C**), IL-1β (**C** and **D**) and IL-6 **E**, **F** expression levels in the cortex and hippocampus of rat. Data are expressed as mean (M ± SEM) and were evaluated by one-way ANOVA followed by post-hoc Tukey’s test. ^#^*P* < 0.05, ^##^*P* < 0.01 vs. the Con group. *P < 0.05, **P < 0.01 vs. the D-gal group

**Fig. 8:**
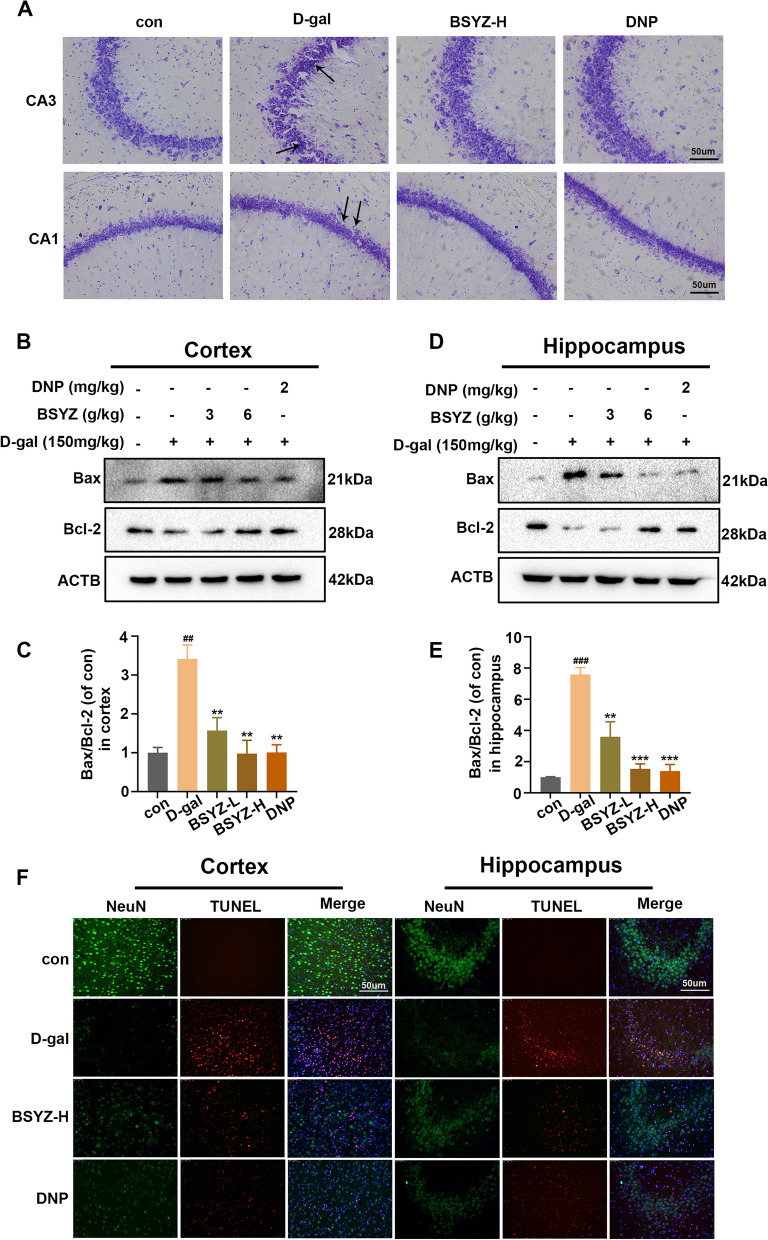
BSYZ alleviates apoptosis, treated by D-gal in rat brain. **A** Representative the result of Nissl (CA1 and CA3 regions) of experimental rat brain. **B**–**E** Representative western blot analysis of Bax and Bcl-2 proteins expression levels in both cortex and hippocampus regions. **F** Representative immunofluorescence images of NeuN (green) and TUNEL (red) of cortex and hippocampus in experimental rat. Data are expressed as mean (M ± SEM) and were evaluated by one-way ANOVA followed by post-hoc Tukey’s test. ^##^*P* < 0.01, ^###^*P* < 0.001 vs. the Con group. **P < 0.01, ***P < 0.001, vs. the D-gal group

Mitochondrial oxidative damage is closely related to neuroinflammation [10] and glial cell activation, which plays an important role in the occurrence and development of neurodegenerative diseases [34]. Therefore, we investigated whether BSYZ display glial cell reactivity. Immunofluorescence analysis further confirmed the presence of glial reactive, which indicated increased GFAP and Iba1 immunoreactivity in D-gal-injected brain slices. Remarkably, BSYZ treatment prominently reduced GFAP and Iba1 immunoreactivity in the cortex and hippocampus brain regions, compared to the D-gal group (Fig. 8A). Since BSYZ displayed a modulation of microglia activity, we investigated whether BSYZ could inhibit various inflammatory mediators, including tumor necrosis factor-alpha (TNF-α), interleukin 1 beta (IL-1β) and interleukin 6 (IL-6) by ELISA assays. We found markedly higher levels of TNF-α, IL-1β and IL-6 in the brains of D-gal-injected rats than the control rats. However, BSYZ treatment reversed the effect of D-gal in TNF-α, IL-1β and IL-6 (Fig. 7). Taken together, BSYZ limits D-gal-induced neuroinflammation in the rat brains.

### BSYZ treatment inhibited apoptotic in D-gal-treated rats

As mitochondrial dysfunction and oxidative stress cause neuronal loss in many neurodegenerative diseases, we, therefore determined whether BSYZ had rescued cell death in D-gal-induced rats [35]. We subjected the brain slices to Nissl staining to access the shape of Nissl bodies. Compared with the control group, the hippocampal Nissl bodies in the D-gal group were disorderly arranged, with blurred edges and hyperchromatic pyknosis, while the number of hippocampal Nissl bodies in the D-Gal + BSYZ-H group increased, with neat arrangement and clear edges (Fig. 8A). What’s more, we analyzed the expression of neuroapoptotic proteins, including Bax and Bcl2, in the cerebral cortex and hippocampus by Western blot. We observed that the Bax/Bcl-2 ratio was significantly up-regulated, leading to D-gal-induced cell death and apoptosis. This situation was reversed in the D-gal + BSYZ co-treatment group, compared with the D-gal group (Fig. 8B–E). Furthermore, NeuN + Tunel double staining was used to evaluate the apoptosis of neurons in the cortex and hippocampus (CA3) area. In the group treated with D-gal alone, the number of NeuN-immunoreative neurons was significantly reduced and the number of Tunel-positive cells was dramatically increased. However, BSYZ treatment remarkedly increased the number of NeuN-immunoreative neurons and decreased the number of Tunel-positive cells compared with the model group (Fig. 8F).

## Discussion

Aging is the major threat component for the improvement of neurodegenerative disorders. D-gal can promote the immoderate generation and accumulation of ROS, thereby promoting the manner of tissue damage and aging, and is a representative of natural growing older animal models. In previous studies, we have uncovered that BSYZ may improve cognitive impairment in AD model mice by controlling oxidative stress, inflammation, and neuronal apoptosis [24, 25]. However, the detailed mechanism of BSYZ on the D-gal induced senescence model has not been investigated yet. In this study, we aim to investigate the potential mechanism of BSYZ on D-gal-induced aging model based on ^1^H-NMR metabolomics analysis. Moreover, this study demonstrated for the first time that BSYZ ameliorated mitochondrial dysfunction-mediated neurodegeneration by means of upregulating the expression of AMPK/Sirt1 through the choline metabolic pathway.

Early research links aging to age-related learning and memory deficits. We employed behavioral tests to assess behavioral changes in experimental aging rat models. We observed a significant improvement in cognitive activity in these artificially aged rats after intragastric administration of BSYZ, suggesting that BSYZ can antagonize D-gal-induced cognitive and behavioral impairments. Another study reported that cognition impairment is largely dependent on the regulation of synaptic plasticity by synaptic proteins [36]. Importantly, the levels of pre- and postsynaptic protein are downregulated in D-gal-induced aging brain [7, 36]. Correspondingly, our results showed that D-gal treatment decreased the expression levels of PSD95 and Syn, whereas BSYZ reversed this trend by significantly upregulating the expression of these memory-related synaptic markers in D-gal-induced rat brain tissue. The neurotrophin BDNF (brain-derived neurotrophic factor) is a member of the neurotrophin family that plays a crucial role in learning and memory [37]. Similarly, we found that BSYZ treatment significantly upregulated the protein expression level of BDNF in D-gal-treated rat brain. Based on these results indicate that BSYZ treatment improves cognitive deficit and synaptic dysfunction.

Metabolomics has emerged as a powerful approach to understand the potential therapeutic mechanisms of TCM herbal decoctions. We employed a ^1^H-NMR-based metabolomic approach and multivariate statistical analysis to better profile changes in metabolite levels and to identify potential target metabolic pathways associated with the aging process after BSYZ treatment. The PCA score map effect confirmed that there was obvious separation in the rat cortical samples of each group, indicating that high-dose BSYZ had a reversal impact on the metabolic profile of rats triggered via D-gal. We used PLS-DA and OPLS-DA score maps to further screen differential metabolites, and found that the effects of BSYZ included an increase in aspartate, betaine, and a decrease in choline. Abnormal accumulation of choline is associated with nerve cell repair, the disassembly of myelin sheaths and neurolipids, and gliosis [38, 39]. Consistent with these studies, the increased level of choline was observed in D-gal-treated aging rats compared with the control rats. However, a higher level of choline in brain was decreased in BSYZ and D-gal co-treatment group. choline can be oxidized to betaine by choline dehydrogenase (CHDH) in mitochondria [40]. Being considered as the final product of choline oxidative metabolism, Betaine, is catalyzed by betaine–homocysteine S-methyltransferase (BHMT) as a highly efficient methyl donor to produce dimethylglycine [41, 42]. Furthermore, betaine can also increase the vitality of BHMT and promote homocysteine metabolism [43]. Accordingly, our western blot results indicated that BSYZ treatment significantly upregulated the proteins expression level of CHDH and BHMT. In addition, the differential metabolites results found that BSYZ could increase betaine levels. These results suggested that BSYZ could regulate choline metabolism in aging model.

Betaine, as the oxidation product of choline [44], is a derivative of the amino acid glycine and a methyl donor [45]. Several research proposed that betaine acts as an endogenous agonist of AMPK and accordingly regulates metabolism [32, 46]. AMPK acts as a key regulator of cellular energy homeostasis and metabolism, sensing intracellular ATP fluctuations, regulating mitochondrial homeostasis [47]and preventing senescence. Furthermore, activation of AMPK also increases intracellular nicotinamide adenine dinucleotide (NAD^+^) concentrations and activates SIRT1 [15, 16]. A reduced in NAD^+^ is hallmarks of oxidative stress-induced cellular senescence. SIRT1 is involved in the regulation of many important biological features such as oxidative stress, energy metabolism, and apoptosis [48]. Another study elucidated that decreased AMPK activity is a cause of the observed decline in mitochondrial function and dynamics during aging [49]. Accumulative evidence illuminated neurons consume most of the total brain energy, so they are inherently dependent on mitochondria and are especially susceptible to conditions such as energy-depleting aging [50]. Mitochondria, a key organelle for the production of oxidants, which are considered key regulators of cell death and survival [51]. Mitochondrial dysfunction leads to bioenergetics deficits and disturbances in cellular redox metabolism [33]. Mitochondrial damage and oxidative stress are viewed early pathogenesis of neurodegenerative diseases [52, 53]. The present study found that BSYZ treatment significantly upregulated the protein expression level of p-AMPK and SIRT1, increased NAD^+^/NADH and ATP levels in comparison to saline-treated D-gal rats, which was the result of BSYZ regulate mitochondrial energy metabolism. It is worth to noting that the oxidation of choline to betaine causes NAD^+^ to accept electrons to produce NADH, reducing the NAD^+^/NADH ratio. However, our results show that BSYZ significantly increased the ratio of NAD^+^/NADH and the levels of ATP. Therefore, we believe that this is most likely because BSYZ treatment maintained normal mitochondrial function, depleted NADH to increase the levels of ATP and NAD^+^. Meanwhile, our BSYZ treated rats revealed a rise in brain endogenous anti-oxidative ability. They displayed significantly reduced SOD and MDA production, as well as enhanced level of GSH in the cortex and hippocampus regions. These results collectively demonstrated BSYZ stimulation of AMPK/SIRT1 may play an important role in alleviating mitochondrial damage and oxidative stress accompanying neurodegeneration in D-gal-induced rats.

Redox control may be a bidirectional link between energy metabolism and inflammatory responses in the brain [54]. Neuroinflammation is frequently observed in neurodegenerative diseases and aging, including microglia activation and inflammatory cytokine production [34]. In aging or neurodegenerative pathological conditions, changes in the function and activity of microglia and astrocytes provoke immune and participate immune responses and participate in brain inflammatory responses [55]. Our D-gal-induced rats showed microglia and astrocytes cell activation and displayed elevated levels of inflammatory mediators, including TNF-α, IL-1β and IL-6. However, BSYZ ameliorated inflammatory response in D-gal model. These findings indicated that BSYZ can counteract D-gal-induced neuroinflammation by inhibiting the activation of glial cells and neuroinflammatory mediators.

Mitochondrial oxidative damage and neuroinflammation are likely to instigate neuronal cell death, and even leading to neurodegeneration [53]. Consistently, Nissl staining and NeuN + Tunel double staining indicated BSYZ + D-gal cotreatment significantly reduced the number of Tunel-positive neuronal cells and increased the number of surviving neurons compare with D-gal-treated rats. The apoptosis pathway is mainly regulated Bcl-2 proteins family, which impacts mitochondria to combine death and survival signals [56]. As a key factor in anti-apoptosis, Bcl-2 protein can effectively prevent peroxidation-induced apoptosis [57]. On the other hand, Bax protein is thought to accelerate cell apoptosis, together with Bcl-2, regulating cell survival or death [58]. Our results presented that BSYZ intervention significantly decreased Bax/Bcl-2 ratio. These data revealed that BSYZ treatment exerted an antiapoptotic effect in D-gal-induced neurodegeneration.

## Conclusions

**Fig. 9:**
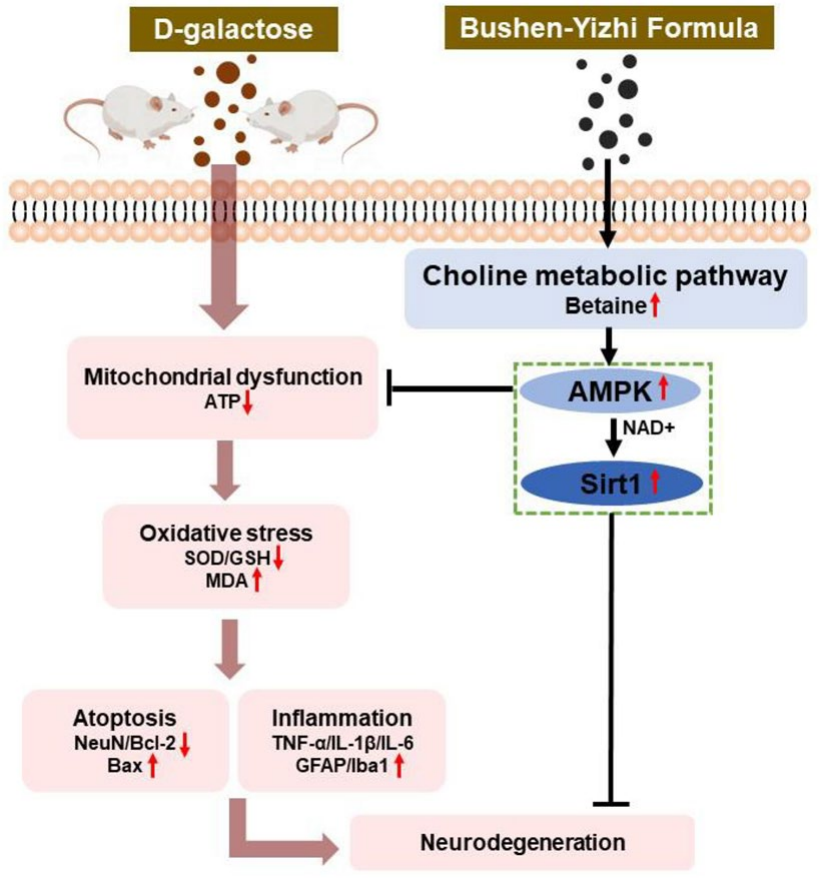
The graphic represents the possible neuroprotective mechanism of Bushen Yizhi formula against D-gal-induced neurodegeneration

We conclude, based on a ^1^H-NMR-based metabolomic approach and multivariate statistical analysis, that BSYZ upregulated AMPK/SIRT1 pathway associated with choline metabolism. In addition, we demonstrate that BSYZ treatment effectively abrogated D-gal inhibition of p-AMPK activity-induced mitochondrial dysfunction, oxidative stress, neuroinflammation, neuroapoptosis, synaptic dysfunction, neurobehavioral impairment in D-gal-treated rats. A proposed simple schema to explain the potential therapeutic effect of BSYZ, is depicted in Fig. 9.

## Data Availability

All data in this study are available from the corresponding author upon reasonable request.

## Abbreviations

BSYZ: Bushen-Yizhi formula
BSYZ-L: Bushen-Yizhi low dose
BSYZ-H: Bushen-Yizhi high dose
D-gal: D-galactose
DNP: Donepezil
AD: Alzheimer’s disease
MWM: Morris water maze test
OFT: Open field test
NOR: Novel object recognition task
CHDH: Choline dehydrogenase
BHMT: Betaine-homocysteine S-methyltransferase
PSD95: Postsynapticdensity 95
SYN: Synaptophysin
BDNF: Brain-derived neurotrophic factor
MDA: Malondialdehyde
SOD: Superoxide dismutase
GSH: Reduced glutathione
TNF-α: Tumor necrosis factor-α
IL-1β: Interleukin-1β
AMPK: Adenosine 5’-monophosphate (AMP)-activated protein kinase
Sirt1: Sirtuin 1
Bax: Bcl-2 Assaciated X protein
Bcl-2: B-cell lymphoma-2
